# Associations of recommended food score and physical performance in Korean elderly

**DOI:** 10.1186/s12889-019-6457-2

**Published:** 2019-01-30

**Authors:** Gyeo Woon Jeong, You Jin Kim, Saejong Park, Hyesook Kim, Oran Kwon

**Affiliations:** 1 Department of Clinical Nutrition Science, The Graduate School of Clinical Health Sciences, Ewha Womans University, Seoul, Republic of Korea; 2 Department of Nutrition, Ewha Womans University Mokdong Hospital, Seoul, Republic of Korea; 3 Department of Nutritional Science and Food Management, Ewha Womans University, 52, Ewhayeodae-gil, Seodaemun-gu, Seoul, 03760 Republic of Korea; 4 Department of Sport Science, Korea Institute of Sport Science, Seoul, Republic of Korea

**Keywords:** Recommended food score, Diet quality, Hand grip strength, Physical performance, Elderly

## Abstract

**Background:**

A single nutrient or dietary pattern has been associated with physical performance. However, little is still known about the association of overall dietary quality with physical performance. This study aimed to investigate the link between the recommended food score (RFS), defined as an indicator of overall diet quality, and physical performance among the Korean elderly aged over 65 years.

**Methods:**

The study subjects consisted of 622 participants (294 men and 328 women) aged over 65 years from the 2014–2015 National Fitness Award project.

**Results:**

The mean value of RFS was higher in elderly women (30.7 ± 7.6) than elderly men (29.5 ± 8.8), but the difference was only marginally significant (*P* = 0.065). In elderly women, multiple regression linear models, adjusted for potential confounders, showed that RFS was positively related to absolute hand grip strength (kg) (ß = 0.066, 95% CI = 0.010 to 0.122) and relative hand grip strength (%) (ß =0.109, 95% CI = 0.016 to 0.201); other physical performance indicators did not show any association with RFS. In elderly men, none of the physical performance indexes were associated with RFS.

**Conclusions:**

These results suggest that a better overall diet quality may be associated with improved grip strength among elderly women in Korea.

## Background

According to resident registration population statistics of the Ministry of the Interior and Safety [1], in 2017, South Korea was categorized as an ‘aged society’. This term is used to define a population, in a country or region, where the proportion of seniors aged over 65 exceeds 14%. South Korea has become one of the most rapidly aging nations because of a sharp decline in the general fertility rate and an increase in life expectancy at birth. This trend has given rise to many problems associated with the elderly [2, 3]. This population is at high risk of health-related issues associated with frailty [4] and of various diseases, including chronic diseases, which lead to increased medical service costs and a decrease in quality of life [5].

Physical performance is important for quality of life, and consists of a healthy body condition, and the capability of dealing with stress and handling social life [6, 7]. Maintaining physical performance in old age is crucial for healthy aging. Poor physical performance in later life has serious health consequences for individuals regarding disability [8, 9], loss of independence [10], as well as mortality [11], and can lead to an increased need for healthcare and social care [12]. However, despite this importance, there is currently a lack of understanding of the factors that affect physical performance.

There is some evidence that physical performance is associated with lifestyle behaviors, including diet [13, 14], as well as non-modifiable factors, such as age and gender [15]. Several studies have reported a positive link between physical performance and intake of specific single nutrients, including protein [16, 17], vitamin D [18–20], dietary fiber [21], antioxidative nutrients like β-carotene, and vitamins E and C [22–24]. Conversely, relatively little is documented about the relation between whole diet and overall physical performance. The few existing investigations have shown that adherence to a Mediterranean-style diet is positively related with physical performance, including mobility and walking speed in older adults [25, 26]. In addition, it has been observed that the healthiest dietary pattern, which means eating the required amounts of fruits, fish/seafood, eggs, nuts and whole grains, may improve physical performance in those aged over 85 years [27].

Some recent literature has focused on the connection between physical performance and overall dietary quality indicators, such as the Health Eating Index (HEI) score [28] or Nordic Diet Score (NDS) [29]. Previous studies conducted in the United States [28] and Finland [29] have shown that a higher dietary quality coexists with better physical performance in the elderly. However, the indicators used in these studies are insufficient to examine the same phenomenon in Korean seniors, because food culture and food source are different.

Most studies on the relationship between diet and physical performance in Koreans have been conducted on adolescents [30], college students [31], or female adults [13, 14, 32]. Although it is possible that overall dietary quality is related to better physical performance, and that better dietary quality aids health and nutrition promotion among elderly people, to the best of our knowledge, no studies have investigated these associations in elderly Korean people. Considering the rapid rise in the aging population in Korea, it is urgent to explore the connection between overall dietary quality and physical performance in the elderly population. Most dietary quality indices are based on food groups and nutrients, whereas recommended food scores (RFS) are based on the foods and food groups recommended in the current American dietary guidelines. RFS is a relatively simple method to assess overall diet. Therefore, this study assessed the overall diet quality by using RFS as an indicator of evaluating dietary quality adapted to the Korean diet [33], and examined the association between RFS and physical performance in men and women aged over 65 years.

## Methods

### Study design and participants

This study was performed within the framework of a prospective, matched case-control study designed to develop criteria referencing health-related fitness standards for the National Fitness Award. The National Fitness Award project is a large-scale national project that the Korea Institute of Sports Science has been conducting since 2012 with the support of the Ministry of Culture, Sports, and Tourism. Adults and seniors have been recruited since 2014, and the present study was based on elderly people (aged 65 or older) who participated in the project in 2014–2015 [34]. Among a total of 1501 participants, we excluded those who had missing data for RFS (*n* = 166), physical performances (*n* = 265), and the covariates (*n* = 84). We also excluded subjects who had musculoskeletal disorders (*n* = 364), such as osteoarthritis, osteoporosis, backache, sciatic neuralgia, and herniated cervical disc, based on the fact that these subjects were also excluded in other studies that examined the association between diet and physical performance [16, 35, 36]. Finally, 622 subjects (294 men and 328 women) aged over 65 years were included in the analysis. The present study was conducted according to the guidelines laid down in the Declaration of Helsinki, and all subjects provided a written informed consent. The study protocols were approved by the Institutional Review Boards at the Korea Institute of Sport Science and Ewha Womans University.

### General characteristics, anthropometric measurement, and socioeconomic characteristics

Detailed information about data collection methods has been reported elsewhere [37]. All of the subjects were interviewed by trained interviewers, to obtain general information on demographic and socioeconomic characteristics, medical history, and health-related behaviors, including age, family income, education, marital status, smoking behavior, alcohol consumption, and RFS. Standing height was measured to the nearest 0.1 cm with a stadiometer (Seca, Seca Corporation, Columbia, MD, USA). Body weight was measured to the nearest 0.1 kg by using an electronic weight scale (Inbody 720, Biospace, Seoul, Korea). Body mass index (BMI) was calculated as weight divided by the square of the height (kg/m^2^). The body fat percentage was estimated by the eight-polar bioelectrical impedance frequencies (Inbody 720, Biospace, Seoul, Korea). Physical activity was assessed through a Korean version of the international physical activity questionnaire (IPAQ) short form [38]. Eleven items of physical activity identified the total minutes of activity in the last 7 days spent on inactivity, walking, and moderate and vigorous-intensity physical activity. Responses were converted to metabolic equivalent task hour per week (MET-hr/wk): walking = 3.3 METs × day × hr.; moderate PA = 4.0 METs × day × hr., and vigorous PA = 8.0 METs × day × hr., where PA is physical activity [39].

### Recommended food score

The recommended food score (RFS) was used to measure overall diet quality. The RFS is a food-based tally, which evaluates the consumption of foods considered to be consistent with existing dietary guidelines, developed by Kant et al [40]. In the current study, the modified RFS proposed by Kim et al. [33] was used because it is appropriate for the Korean diet, which includes food items, such as mixed grains, beans, vegetables, seaweed, fruits, seafood, dairy products, and nuts. The food items and their corresponding points for the RFS were as follows: daily frequency of meals (1), grains (1), legumes (4), vegetables (17), seaweeds (2), fruits (12), fish (5), dairy products (3), nuts (1) and tea (1). A total of 46 foods or food groups consistent with the recommended food groups were selected and one response for ‘daily frequency of meals’ was used to calculate the RFS. Participants were allocated one point for each recommended food or regular eating pattern (three meals per day) if they consumed the food at least once per week. The score ranged from 0 to 47 points, and higher scores imply a greater diet quality.

### Assessment of physical performances

Physical performances were assessed by a physical performance test of the elderly at the national fitness award. The national physical fitness test parameters yielded significantly consistent results, with reliability ranging from 0.62–0.93 [41]. All parameters were measured by certified, professional health and fitness instructors. Four assessments were conducted, including a 2-min step test (2MST), timed up and go test (TUG), figure-of-8 walk test (F8W), hand grip strength test, and arm curl test. The concrete methods of physical performances are as follows:

(1) 2MST (rep·120 s^− 1^): The participant stands up straight next to the wall, while the level corresponding to midway between the patella and iliac chest. The 2-min step requires the participants to march in place for 2 min, lifting the knees to a marked target on the wall, which is set at each individual’s midpoint between their hip and knee. The number of times the right knee met the marked target was counted. An increased step count within the 2 min was reflective of a greater cardiopulmonary endurance.

(2) TUG (sec): This test assesses a person’s mobility and balance. Participants were instructed to complete the course at their usual pace, by standing up without any support, walking 3 m and around a cone/mark on the floor, walking back to the chair, and then sitting down again. The time that the participant needed to complete the test was recorded. A longer time indicates worse balance and mobility performance.

(3) F8W (sec): The F8W, as a measure of walking skill, evaluates movement control and planning during walking [42]. Participants were instructed to stand midway between two cones placed about 5 ft (1.5 m) apart, start walking at their usual pace in a figure-of-eight walking path around the cones and stop upon returning to the starting position. Timing began when the subject started to take the first step and ended when both of their feet were back in the starting position.

(4) Hand grip strength (kg, %): Hand grip strength was measured using a hand dynamometer (GRIP-D 5101, Takei, Niigata, Japan) placed between the fingers and palm at the base of the thumb in one hand, with the arm extended to the side and squeezed with maximum force. Participants were instructed to stand in the upright position, keep their arms straight, maintain a 15° angle between the arm and torso, and squeeze the dynamometer for 5 s at maximal effort. After both the left and right hand were performed twice, the highest value was recorded to the nearest 0.1 kg. For relative hand grip strength (%), the absolute hand grip strength (kg) was divided by the participant’s body weight (kg) and then multiplied by 100.

(5) Arm curl test (frequency): The arm curl test assesses upper body strength and endurance. The participant was instructed to sit on the chair, hold the weight in the dominant hand, with palm facing towards the body, and the arm in a positioned vertically down beside the chair. Then, the participant was to brace the upper arm against the body so that only the lower arm was moving, and curl the arm up through a full range of motion, gradually performing elbow flexion with supination. The score is the mean of the total number of left and right arm curls performed in 30 s. The frequency of biceps curls (left and right sides) was recorded.

### Statistical analysis

Characteristics of participants were presented as means and standard deviation (SD) for continuous variables, and a number and percentage for categorical variables. Differences in means and the distribution of general characteristics between men and women were analyzed using the Student’s *t*-test and chi-square test. The differences in distribution of categorical variables (income, education, smoking, drinking) between men and women were analyzed using the Chi-Square test. Student’s *t*-test was used to compare continuous variables (age, height, weight, BMI, body fat, physical activity (MET-hr/wk), the RFS, and physical performances) between men and women. Pearson’s correlation test was used to analyze correlations between RFS and physical performances. After controlling for potential confounders, multiple regression model analyses were conducted to examine the association between the RFS and physical performances. The covariates were statistically significant in univariate analyses or were known to be risk factors associated with the RFS or physical performance, in existing literature. Model 1 was adjusted for age, body fat percentage, income, education, marital status, smoking, and drinking. Model 2 was adjusted for physical activity (MET-hr/wk), in addition to the adjustments made in Model 1. Multicollinearity between independent variables was checked by performing variance inflation factor (VIF) tests. All statistical analyses were performed using SAS (ver. 9.4, SAS Institute, Cary, NC, USA).

## Results

### General characteristics

The mean age of men was 71.7 ± 4.6 years, and the mean age of women was 71.9 ± 5.2 years. The mean height, weight, and lean body mass were higher in men than women, whereas the mean BMI and body fat percentage were higher in women. Women had a greater mean RFS (30.7 ± 7.6) than men (29.5 ± 8.8), but the difference was not significant. Socioeconomic characteristics and health-related behaviors, such as income, education, marital status, smoking, and drinking, were significantly different between the genders. Among physical performances, the mean TUG (*P* < 0.001) and F8W (*P* < 0.001) in men were faster compared to women, and the mean scores of grip strength (*P* < 0.001) and arm curl test (*P* < 0.001) were higher in men (Table 1).

**Table 1:** General characteristics of the elderly subjects

	Total (*n* = 622)	Men (*n* = 294)	Women (*n* = 328)	
	Mean ± S.D. or n (%)	Mean ± S.D. or n (%)	Mean ± S.D. or n (%)	*P*-value
Age, yrs	71.8 ± 4.9	71.7 ± 4.6	71.9 ± 5.2	0.654
Height, cm	159.0 ± 8.6	166.1 ± 5.6	152.6 ± 5.2	<.0001
Weight, kg	60.5 ± 8.8	65.2 ± 8.0	56.3 ± 7.3	<.0001
BMI, kg/m^2^	23.9 ± 2.7	23.6 ± 2.5	24.2 ± 2.8	0.008
Body fat, %	29.8 ± 7.9	24.2 ± 5.6	34.8 ± 6.0	<.0001
Lean body mass, kg	22.9 ± 4.7	27.0 ± 3.1	19.3 ± 2.3	<.0001
Income, won/mo				0.0002
Less than 2,000,000	287 (46.1)	159 (54.1)	128 (39.0)	
Over 2,000,000	335 (53.9)	135 (45.9)	200 (61.0)	
Education				<.0001
Elementary/middle school	338 (54.3)	115 (39.1)	223 (68.0)	
High school	184 (29.6)	101 (34.4)	83 (25.3)	
College	100 (16.1)	78 (26.5)	22 (6.7)	
Marital status				<.0001
Single	207 (33.3)	42 (14.3)	165 (50.3)	
Marital	415 (66.7)	252 (85.7)	163 (49.7)	
Current smoker, yes	39 (6.3)	36 (12.2)	3 (0.9)	<.0001
Current drinker, yes	235 (37.8)	173 (58.8)	62 (18.9)	<.0001
Physical activity, METs-h/wk	8.7 ± 9.4	8.4 ± 8.3	8.9 ± 10.3	0.535
Recommended food score	30.1 ± 8.2	29.5 ± 8.8	30.7 ± 7.6	0.065
Physical performance				
2-min step test, repㆍ120sec	105.2 ± 23.3	104.5 ± 20.7	105.7 ± 25.5	0.524
Timed up and go test, sec	6.4 ± 1.6	6.0 ± 1.3	6.7 ± 1.8	<.0001
Figure of 8 walk test, sec	27.6 ± 7.0	26.0 ± 5.6	29.1 ± 7.8	<.0001
Grip strength, kg	27.8 ± 8.2	34.5 ± 6.1	21.8 ± 4.1	<.0001
Grip strength, %	45.8 ± 11.0	53.4 ± 9.1	39.0 ± 7.7	<.0001
Arm curl test, frequency	16.8 ± 5.0	18.3 ± 5.2	15.5 ± 4.3	<.0001

S.D., standard deviation; BMI, body mass index; Current drinker was defined as consuming alcohol more than once a month and current smoker was defined as current smoking or cessation of smoking within the past 12 months. Marriage was defined as reporting married or living as married

### Correlation between RFS and physical performances

In women, RFS was positively correlated with absolute hand grip strength (kg) (r = 0.149, *P* = 0.007) and negatively correlated with F8W (r = − 0.113, *P* = 0.041). There was no correlation between RFS and physical performance in men (Table 2).

**Table 2:** Correlation between RFS and physical performances in the elderly subjects

	Men (*n* = 294)		Women (*n* = 328)	
	r	*P*-value	r	*P*-value
2-min step test, repㆍ120sec	0.050	0.390	0.039	0.487
Timed up and go test, sec	−0.034	0.560	−0.064	0.246
Figure of 8 walk test, sec	0.045	0.443	−0.113	0.041
Grip strength, kg	−0.027	0.648	0.149	0.007
Grip strength, %	−0.113	0.052	0.097	0.080
Arm curl test, frequency	− 0.036	0.544	0.056	0.309

### Association between RFS and physical performances

As shown in Table 3, after adjustment for possible confounders (Model 1), multiple regression analysis revealed a positive association between the RFS and grip strength (kg) (ß = 0.063, 95% CI = 0.007 to 0.119) and grip strength (%) (ß =0.105, 95% CI = 0.013 to 0.198) in women; this trend was also observed after further adjustment for physical activity (grip strength (kg) (ß = 0.066, 95% CI = 0.010 to 0.122) and grip strength (%) (ß =0.109, 95% CI = 0.016 to 0.201). Other physical performance indicators did not show any association with RFS. No significant association was observed between the RFS and any of the physical performances in men.

**Table 3:** Association between RFS and physical performances in the elderly subjects

	Men (*n* = 294)			Women (*n* = 328)		
	ß	95% CI	*P*-value	ß	95% CI	*P*-value
2-min step test, repㆍ120sec						
Unadjusted	0.119	−0.153, 0.390	0.390	0.130	−0.237, 0.497	0.487
Model 1	0.086	−0.179, 0.351	0.523	0.020	−0.327, 0.367	0.909
Model 2	0.096	−0.167, 0.358	0.474	0.039	−0.305, 0.384	0.823
Timed up and go test, sec						
Unadjusted	−0.005	−0.021, 0.012	0.560	−0.015	− 0.041, 0.010	0.246
Model 1	−0.005	−0.020, 0.010	0.480	−0.002	− 0.022, 0.019	0.878
Model 2	−0.006	−0.021, 0.009	0.458	−0.002	− 0.023, 0.018	0.816
Figure of 8 walk test, sec						
Unadjusted	0.029	−0.045, 0.103	0.443	−0.116	−0.227, − 0.005	0.041
Model 1	0.016	−0.047, 0.079	0.626	−0.057	−0.148, 0.035	0.222
Model 2	0.014	−0.049, 0.077	0.660	−0.059	−0.149, 0.031	0.197
Grip strength, kg						
Unadjusted	−0.019	−0.098, 0.061	0.648	0.081	0.022, 0.140	0.007
Model 1	0.005	−0.070, 0.079	0.905	0.063	0.007, 0.119	0.027
Model 2	0.005	−0.070, 0.080	0.900	0.066	0.010, 0.122	0.022
Grip strength, %						
Unadjusted	−0.118	−0.236, 0.001	0.052	0.098	−0.012, 0.209	0.080
Model 1	−0.013	−0.112, 0.086	0.799	0.105	0.013, 0.198	0.026
Model 2	−0.014	−0.113 0.085	0.780	0.109	0.016, 0.201	0.022
Arm curl test, frequency						
Unadjusted	−0.021	− 0.089, 0.047	0.544	0.032	−0.030, 0.095	0.309
Model 1	−0.025	−0.090, 0.041	0.459	0.013	−0.048, 0.075	0.668
Model 2	−0.022	−0.087, 0.042	0.499	0.014	−0.046, 0.075	0.639

Model 1: adjusted for age, body fat percentage, smoking, drinking, education, income, marital status

Model 2: adjusted for age, body fat percentage, smoking, drinking, education, income, marital status, physical activity

## Discussion

This study found that overall diet quality (RFS) was positively linked to grip strength among elderly Korean women, but not elderly men. To the best of our knowledge, this is the first study showing a positive association between the RFS and grip strength in elderly Korean subjects.

The RFS was used as a simple tool to evaluate the quality of the overall diet, in our study. However, pre-existing studies have used various other tools to determine the overall diet scores. Beibei et al. [28] showed that a higher HEI score is positively linked to physical performance in adults aged over 60 years. A 10-year cohort study by Mia-Maria et al. [29] reported a significant positive association between NDS and physical performance in women. Especially, a high consumption of fruit and berries, cereals, and alcohol, among NDS food items, was positively related to the overall physical performance in women [29]. It was presumed that the results of the current research would be similar to those mentioned in the literature because the components of the RFS, including fruits and vegetables, fish, dairy products, and grains, are similar to food components in the tools mentioned above. In this study, the mean RFS of men and women was 30.7 and 29.5, respectively, of a total 47 points, and the mean RFS for women was marginally significantly higher than that for men. The RFS values of this study’s subjects were somewhat higher than those of other countries, although the total RFS points were different because there is a different way of calculating RFS according to the dietary characteristics of the people of each country. In a study of Australian men and women aged 55–65 years [43], mean RFSs were 23 and 26.6, respectively, which was lower than the RFS of our study subjects. However, in Australia, the maximum RFS is 49, which is two points higher than that used in our study. In the US Nurses’ Health Study [44], the mean RFS of women aged 43–69 years was 18 out of a total 51 points, which was significantly lower than that of our subjects. This suggests that the dietary quality of Korean people, as calculated using RFS, is higher than in other countries.

Akin to overall diet quality, several researchers have documented a link between dietary pattern and physical performance in the elderly. A prospective study by Antoneta et al. [27] found that a healthy dietary pattern, consisting of high intakes of fish, fruits, nuts, cereal, vegetables, dairy products, and oils rich in unsaturated fatty acids, has a prolonged effect on better age-related physical performance and reduces adverse health outcomes in the elderly. Moreover, physical performance, including mobility and walking speed, was greater in those with higher adherence to the Mediterranean dietary pattern in cohort studies [25, 26]. Given that dietary pattern and the RFS are related, a superior RFS may mean that sufficient food intake, consisting of healthy nutrients, is related to physical performance and various other health benefits.

There are several mechanisms explaining the association between physical performance and the RFS. The RFSs in this study were calculated using information about the overall intake of fruits, vegetables, whole grains, milk products, legumes, fish, and nuts. These are food items that contain a substantial amount of complex carbohydrates, unsaturated fat, fiber, and antioxidants, such as vitamins, minerals, and phytonutrients [33]. According to preceding studies, antioxidants have an important role in increasing skeletal muscle repair and a protective function against the age-dependent increase in oxidative stress [28, 33, 45]. Oxidative stress is considered to be the common factor underlying chronic diseases, such as diabetes and cardiovascular disease, which, in turn, leads to poor physical performance and muscle function [28, 45]. According to Kim et al. [33], a higher RFS indicated a high consumption of energy, macronutrients, and antioxidants, and was associated with muscle function, as well as a decline in oxidative stress. Therefore, it could be suggested that a higher RFS resulted in beneficial effects on physical performance through, at least in part, a reduction in oxidative stress.

Another possible mechanism to explain the connection between physical performance and the RFS may be related to the positive effects of dietary fiber on systemic inflammation [21] via the reduction of the serum high sensitivity C-reactive protein level. Systemic inflammation contributes to poor physical performance. A high fiber intake can also provide beneficial metabolic effects, such as an attenuation of hyperglycemia, improved insulin sensitivity, and hypercholesterolemia, which, in turn, are related to enhanced physical performance. Several food items of the RFS, such as whole grains, fruits, and vegetables are rich in dietary fiber, which may have contributed to establishing physical exercise benefits.

In the present study, among physical performance measures, grip strength was particularly associated with the RFS in elderly women. We do not know the exact reason why other physical performance indicators were not related to RFS. However, RFS is a dietary indicator related to antioxidant capacity, and grip strength is a major determinant of sarcopenia or frailty associated with oxidative stress among the various physical performance measures. It is thus presumed that grip strength is the most sensitive index among physical performance measures associated to dietary factors. In recent years, there has been accumulating scientific evidence of a significant inverse relationship between hand grip strength and total mortality [46], chronic disease morbidity [47], and mortality [48], even after adjustment for major risk factors and cardiorespiratory function [49]. Some studies conducted on representative national data of the Korean population revealed a trend toward a lower prevalence of hypertension [50] and improved health-related quality of life with increased grip strength [51, 52]. Moreover, a study conducted in Korea suggested grip strength as a clinical marker for muscle strength, to diagnose sarcopenia and that the cut-off values for grip strength might be useful as reference values when classifying sarcopenia [53].

Hand grip strength is used to assess muscle strength, and it is also an outcome predictor and marker of nutritional status [36, 54]. According to several studies related to hand grip strength and nutrition, nutritional status has an important role in muscle strength because muscle function reacts quickly to dietary variations [15] and grip strength may be easily influenced by diet [36, 54]. Robinson et al. [55] noted that grip strength was positively influenced by a high prudent dietary pattern score, characterized by a high consumption of fruit, vegetables, wholemeal cereals, and fatty fish. Furthermore, an adequate intake of dietary protein, antioxidants, and vitamin D was related to a decline in oxidative stress, anti-inflammation, and maintenance of muscle function, resulting in a greater grip strength [55]. In this context, grip strength is related to diet, the prevalence of various diseases, and the health-related quality of life. Thus, a better dietary quality is important for improved muscle strength and prevention of a range of diseases.

In this study, it was observed that RFS was associated with physical performance, especially grip strength in elderly women but not men. Similar gender differences were demonstrated by Mia-Maria et al. [29]. They observed that a healthy Nordic diet was related to better overall physical performance in older Finnish women but not in men. Martin et al. [56] found that the associations between single nutrients and foods (rather than a diet quality index) and physical performance exist in a UK population of community-dwelling older women but not in men. Mishra et al. [57] also reported that higher daily protein intake was positively associated with grip strength in US women but not in men. The reasons for this gender difference are unclear, but they may be explained by the difference in absolute value in grip strength and muscle mass [58, 59] between men and women or the degree of decrease in grip strength and muscle mass [58, 60] with increasing age. This may be related to sex differences in hormonal factors [60, 61]. It has been reported that compared to women, a rapid age-related decline in physical function and grip strength was observed in men despite their higher initial levels of muscle strength score and grip strength [58]. Doherty demonstrated that the greater loss of muscle mass observed in men may be related to declines in growth hormone and androgens with aging [60]. Taekema et al. reported that the significant relationship between insulin-like growth factor 1 (IGF1) levels and muscle strength found in women but not in men suggests a gender-specific influence of IGF1 on muscle strength [61]. Further research is needed to determine the exact mechanism to explain the effect of gender disparity on the association between RFS and grip strength.

The present study found that a high RFS was associated with grip strength, even after adjusting for physical activity. According to several studies, physical activity is known to be positively associated with physical performance in the elderly [62, 63]. Conversely, Beibei et al. [28] noticed that an association between HEI-2005 scores and physical performance in the elderly was attenuated and no longer statistically significant after adjusting for physical activity. Similarly, another study showed that the link between NDS and physical performance measures was attenuated after adjusting for various factors, including physical activity [29].

The limitations of this study are as follows: first, we cannot determine a causal association between the RFS and physical performance because our research is a cross-sectional study design. Further longitudinal studies are required, to establish the causal relationship between diet quality and physical performance. Second, this study only used the RFS to assess the diet quality, and no instruments were used for evaluating the amount of intake or nutrients. The RFS, in this study, was based on a daily frequency of meals or food frequency, which might have resulted in under- or overestimating of an individual’s intakes. However, a previous study [33] observed that energy intake and the intake of macro- and micronutrients, except vitamins A and C, were higher in subjects with a higher RFS. Therefore, a high RFS may be included mean of high intake of energy and nutrients. To our knowledge, despite these limitations, the current study is the first to observe that the RFS was positively associated with grip strength among elderly women in Korea.

## Conclusions

In conclusion, we found that a higher RFS was associated with an improved hand grip strength among Korean elderly, particularly in women. Further prospective studies are warranted to verify the association between RFS and hand grip strength in elderly Korean populations.

## Abbreviations

2MST: 2-min step test;
F8W: figure of 8 walk test
HEI: Healthy Eating Index;
MET: metabolic equivalent task;
NDS: Nordic Diet Score
RFS: recommended food score;
TUG: timed up and go test;
